# In utero origin of sex-related differences in future cardiovascular disease

**DOI:** 10.1186/s13293-016-0108-4

**Published:** 2016-10-13

**Authors:** Sarah Schalekamp-Timmermans, Jerome Cornette, Albert Hofman, Willem A. Helbing, Vincent W. V. Jaddoe, Eric A. P. Steegers, Bero O. Verburg

**Affiliations:** 1 Department of Obstetrics and Gynecology, Erasmus MC University Medical Centre Rotterdam, P.O. Box 2040, 3000 CA Rotterdam, The Netherlands; 2 Department of Epidemiology, Erasmus MC University Medical Centre Rotterdam, P.O. Box 2040, 3000 CA Rotterdam, The Netherlands; 3 Department of Pediatrics, Erasmus MC University Medical Centre Rotterdam, P.O. Box 2040, 3000 CA Rotterdam, The Netherlands

**Keywords:** Sex-specific, Pregnancy, Women, Men, Hemodynamics, Pregnancy

## Abstract

**Background:**

There are sex differences in the risk of development of cardiovascular disease (CVD). According to the developmental origins of health and disease paradigm (DOHaD), CVD originates in fetal life. This study examines fetal sex differences in cardiovascular development in utero.

**Methods:**

In 1028 pregnant women, we assessed fetal circulation using pulsed wave Doppler examinations between 28 and 34 weeks gestation. To test associations between fetal sex and fetal circulation measurements, linear regression models were used adjusting for fetal size, gestational age, and fetal heart rate.

**Results:**

A higher pulsatility index in the ductus venosus was observed in male fetuses compared to female fetuses (difference 0.02, 95 % CI 0.01; 0.05) with a lower E/A ratio of the tricuspid (difference −0.01, 95 % CI −0.03; −0.00) and mitral (difference −0.02, 95 % CI −0.03; −0.01) valves. This was mainly determined by differences in the E wave of the tricuspid and mitral valves (differences −1.02, 95 % CI −1.81; −0.24 and −1.28, 95 % CI −2.11; −0.46, respectively). Also in males, a lower peak systolic velocity was seen in the pulmonary artery (difference −1.33, 95 % CI −2.63; −0.03) with a similar lower trend regarding peak systolic velocity in the ascending aorta.

**Conclusions:**

Male fetuses exhibit an increased preload and reduced afterload conditions compared to females. While it is difficult to relate these measurements to exact cardiac function, our findings strongly suggest that the known differences in cardiovascular performance between the sexes already start in utero.

## Background

Cardiovascular disease (CVD) is the number one cause of death globally [1]. CVD develops over decades, but according to the theory of developmental plasticity, its cornerstone is already laid during fetal life [2].

There are known differences between men and women in age-dependent onset, severity, symptoms, and outcomes of CVD [3]. Women are protected from most cardiovascular events compared to men until after menopause. However after menopause, there is a clear shift in this risk. Men are furthermore thought to be at risk of cardiovascular disease at earlier ages than women. At present, changes in the hormonal environment are thought to account for these differences. However, other mechanisms including, for example, the function of the vascular endothelium and smooth muscles between men and women might also be involved [3].

Previously, it was shown that fetal and early life cardiovascular adaptations exhibit important long-term consequences for the future health of the individual. Left ventricular hypertrophy is a strong and independent risk factor of cardiovascular morbidity and mortality in adulthood, and it has been shown that left ventricular mass tracks from fetal life through childhood into adulthood [4]. Similarly, it has been reported that elevated blood pressure tracks from childhood into adulthood with eventual progression into essential hypertension [5].

When it comes to prevention of sex-specific differences in CVD, greater knowledge of the pathophysiological mechanisms involved is essential. With this study, we want to examine the hypothesis that the observed sex-specific differences in cardiovascular performance in later life might already start in utero. We will assess this by exploring fetal sex-specific differences in cardiovascular development and hemodynamic patterns in utero.

## Methods

### Aims

We aim to examine fetal sex-specific differences in cardiovascular development and hemodynamic patterns in utero.

### Design, setting, and participants

This study was embedded in the Generation R Study, a prospective cohort study from fetal life onwards [6]. Detailed assessments of fetal growth and development were conducted in a subgroup of 1232 Dutch mothers and children, referred to as the Generation R Focus Study [7, 8]. Of all approached mothers, 80 % were enrolled in this subgroup. This subgroup is ethnical homogeneous to exclude possible confounding or effect modification by ethnicity. For the present study, fetal circulation variables were assessed between 28 and 34 weeks gestation. Women with twin pregnancies (*n* = 15), perinatal deaths (*n* = 2), pregnant women with a single umbilical artery (*n* = 8), pregnant women with a comorbidity including maternal congenital cardiac disease and maternal chronic hypertension (*n* = 26), and pregnant women with missing data on either one of the abovementioned variables (*n* = 153) were excluded from analyses. No major fetal cardiac anomalies were present other than small ventricular septum defects (*n* = 3). Present analyses were performed in a total of 1028 subjects. Written informed consent was obtained from all participants. The study has been approved by the Medical Ethics Committee of the Erasmus Medical Center, Rotterdam. The dataset supporting the conclusions of this article is available upon signed request via http://generationr.nl.

### Fetal biometry and Doppler measurements

In a research setting at a regional health facility in the center of Rotterdam, ultrasound examinations assessing gestational age and fetal growth characteristics were carried out in early (<18 weeks gestation), mid (18–25 weeks gestation), and late pregnancy (>25 weeks gestation) [9]. Estimated fetal weight was calculated with the formula by Hadlock using head circumference, abdominal circumference, and femur length [10].

Fetal circulation variables were assessed by pulsed wave Doppler between 28 and 34 weeks gestation in the Generation R Focus Group. For all Doppler measurements, color imaging was used to optimize the placement of the pulsed wave Doppler gate. The insonation angle was kept as close to 0° as possible and always below 20°. The sample volume was adjusted to cover the entire vessel. For each measurement, three consecutive uniform waveforms were recorded by pulsed Doppler ultrasound, during fetal apnea and without fetal movement. The mean of three measurements was used for further analysis. Three experienced sonographers performed all measurements. Intra- and interobserver reproducibility has been reported showing high intraclass correlation coefficient values with corresponding low coefficient of variation values representing adequate reproducibility for all Doppler measurements [7, 8]. Umbilical artery pulsatility index (PI) was measured in a free-floating loop of the umbilical cord. Middle cerebral artery Doppler measurements were performed with color Doppler visualization of the circle of Willis in the fetal brain, and the flow velocity waveforms were obtained in the proximal part of the cerebral arteries.

Diastolic function was assessed by evaluating flow velocity waveforms at the level of the mitral and tricuspid valves. These were recorded from the apical four-chamber view of the fetal heart, placing the sample volume just below the atrioventricular valves (pasileo). Color Doppler visualization of the blood flow allowed us to align the Doppler beam in the direction of the blood flow. The E wave represents early passive ventricular filling and the A wave the contribution of active atrial contraction to ventricular filling. Peak E and A velocities were recorded. The E/A ratio was calculated.

Outflow flow velocity waveforms from the aorta and pulmonary artery were recorded from the five-chamber view and the short-axis view of the fetal heart just above the semilunar valves, respectively. Peak systolic velocity (PSV), time velocity integral (TVI), fetal heart rate, and the inner diameter during systole of both arteries were recorded. Left and right cardiac output was calculated in milliliters per minute by multiplying the vessel area × TVI × fetal heart rate. Combined cardiac output was calculated by adding left and right ventricular output. Finally, flow assessment at the level of the ductus venosus was carried out in both a transversal or parasagittal oblique scanning plane of the fetal abdomen immediately after the origin of the ductus from the umbilical vein. The venous pulsatility index (PIV) was assessed. All ultrasound examinations were performed using an ATL-Philips® Model HDI 5000 (Seattle, WA, USA) equipped with a 5.0-MHz, high-frequency curved array transducer.

### Covariables

Data regarding maternal age, parity, comorbidity including maternal congenital cardiac disease and chronic hypertension, educational level, smoking habits, and folic acid use were obtained through self-administered questionnaires (response rate 93 %) [6]. At enrollment, maternal height and weight were measured to calculate body mass index (BMI, kg/m^2^). Information on pregnancy outcomes was obtained from medical records completed by community midwives and obstetricians.

### Statistical analysis

To test differences in subject characteristics between male fetuses and female fetuses, the independent sample *t* test, the Mann–Whitney U test, and chi-square test were used. Secondly, we performed the independent sample *t* test and Mann–Whitney U test to test fetal sex-specific differences in the fetal cardiac and circulation Doppler measurements. Next, the associations between fetal sex and the fetal cardiac and circulation measurements were assessed using multiple linear regression models adjusted for fetal heart rate, fetal weight, and gestational age at the performed measurement. Regression coefficients were calculated with their 95 % confidence intervals (95 % CI). All statistical analyses were performed using Statistical Package of Social Sciences version 20.0 for Windows (SPSS Inc., Chicago, IL, USA).

## Results

A total of 1028 subjects were included in the study of which 532 were male fetuses (51.8 %) and 496 were female fetuses (48.2 %) (Table 1). At 30 weeks gestation, male fetuses were slightly larger than female fetuses (estimated fetal weight of 1632 g [standard deviation (SD) 255 g] versus 1582 g [SD 255 g], respectively). Similar findings at birth were observed with an average difference of 80 g (birth weight 3571 g [SD 529 g] versus 3490 g [SD 540 g], respectively). No other differences in baseline characteristics were observed.

**Table 1:** Subject characteristics (*n* = 1028)

		Fetal sex		
	Total	Female fetuses	Male fetuses	
	*N* = 1028	*n* = 496	*n* = 532	*p* value
Gestational age enrolment (weeks)	12.9 (6.9–24.5)	12.9 (7.4–24.5)	12.9 (6.9–23.7)	NS
Maternal age (years)	31.5 ± 4.1	31.6 ± 4.0	31.5 ± 4.2	NS
Parity (% 0)	61.2	60.9	61.5	NS
Educational level (% university/college)	63.3	75.7	62.5	NS
BMI intake (kg/m^2^)	23.1 (16.7–43.3)	23.3 (17.9–41.9)	23.0 (16.7–43.3)	NS
Smoking during pregnancy (% No)	75.7	75.8	75.7	NS
Preconception folic acid use (% Yes)	60.9	61.2	60.5	NS
Gestational age third trimester (weeks)	30.4 (27.4–35.1)	30.3 (27.4–35.0)	30.4 (27.6–35.1)	NS
Estimated fetal weight (grams)	1607 ± 252	1582 ± 255	1632 ± 255	<0.05
Ductus venosus PIV	0.56 ± 0.17	0.55 ± 0.16	0.57 ± 0.18	<0.05
E wave tricuspid valve	42.9 ± 5.8	43.3 ± 6.0	42.6 ± 5.6	<0.05
A wave tricuspid valve	56.0 ± 7.8	56.3 ± 7.9	55.6 ± 7.7	NS
E/A ratio tricuspid valve	0.77 ± 0.09	0.77 ± 0.09	0.77 ± 0.08	NS
E wave mitral valve	40.0 ± 6.1	40.5 ± 6.3	39.6 ± 5.9	<0.05
A wave mitral valve	51.5 ± 7.7	51.9 ± 7.8	51.1 ± 7.6	<0.05
E/A ratio mitral valve	0.78 ± 0.09	0.79 ± 1.0	0.78 ± 1.0	<0.05
Umbilical artery PI	0.97 ± 0.17	0.99 ± 0.17	0.95 ± 0.17	<0.05
Middle cerebral artery PI	2.0 ± 0.3	2.0 ± 0.3	2.0 ± 0.3	NS
Aorta ascendens PSV (cm/s)	90.9 ± 12.6	91.5 ± 12.4	90.5 ± 12.8	NS
Pulmonary artery PSV (cm/s)	73.7 ± 9.7	74.2 ± 10.0	73.1 ± 9.5	NS
Fetal heart rate (beats/min)	138.5 ± 8.8	139.2 ± 8.2	137.9 ± 9.2	<0.05
Combined cardiac output (ml/min)	1485 ± 389	1481 ± 362	1489 ± 413	NS
Gestational age at birth (weeks)	40.3 (30.4–43.4)	40.3 (30.4–42.7)	40.3 (32.6–43.4)	NS
Birth weight (grams)	3532 ± 542	3490 ± 540	3570 ± 529	<0.05

Values are means (standard deviation ± SD) or medians (range)

*BMI* body mass index, *PI* pulsatility index, *PIV* pulsatility index for veins, *PSV* peak systolic velocity, *NS* not significant

### Preload

Table 2 shows the results of the linear regression analyses. Values are regression coefficients and reflect differences in fetal cardiovascular and circulation measures of male fetuses compared with female fetuses (reference category) in the third trimester of pregnancy (median 30.4 weeks; range 27.4–35.1 weeks). There was a significantly higher pulsatility index of the ductus venosus (difference 0.02, 95 % confidence interval (CI) 0.01; 0.05, *p* < 0.05) with a lower E/A ratio in both the tricuspid (difference −0.01, 95 % CI −0.03; −0.00, *p* < 0.05) and mitral (difference −0.02, 95 % CI −0.03; −0.01, *p* < 0.05) valves in male fetuses. This was mainly determined by significant differences in the E wave of the tricuspid and mitral valves when comparing male fetuses to female fetuses (difference −1.02, 95 % CI −1.81; −0.24 and difference −1.28, 95 % CI −2.11; −0.46, respectively). No significant differences were found in the A waves of the tricuspid and mitral valves between the sexes.

**Table 2:** Fetal cardiovascular and circulation measures

	Fetal sex		
	Female fetuses	Male fetuses	
	*n* = 496	*n* = 532	*p* value
Preload			
Ductus venosus PIV	ref	0.02 (0.01; 0.05)	<0.05
E wave tricuspid valve^a^	ref	−1.02 (−1.81; −0.24)	<0.05
A wave tricuspid valve^a^	ref	−0.49 (−1.55; 0.58)	NS
E/A ratio tricuspid valve^a^	ref	−0.01 (−0.03; −0.00)	<0.05
E wave mitral valve^a^	ref	−1.28 (−2.11; −0.46)	<0.05
A wave mitral valve^a^	ref	−0.55 (−1.59; 0.50)	NS
E/A ratio mitral valve^a^	ref	−0.02 (−0.03; −0.01)	<0.05
Afterload			
Umbilical artery PI	ref	−0.03 (−0.05; −0.01)	<0.05
Middle cerebral artery PI	ref	−0.03 (−0.07; 0.01)	NS
Ascending aorta PSV (cm/s)^a^	ref	−0.94 (−2.60; 0.71)	NS
Pulmonary artery PSV (cm/s)^a^	ref	−1.33 (−2.63; −0.03)	<0.05
Fetal heart rate (beats/min)	ref	−1.71 (−2.90; −0.53)	<0.05
Combined cardiac output (ml/min)^a^	ref	−21.8 (−71.00; 27.38)	NS

Results from multiple linear regression analyses. Values are regression coefficients (95 % confidence interval (CI)) and reflect differences in fetal cardiovascular and circulation measures of male fetuses compared with female fetuses (reference category) in the third trimester of pregnancy (median 30.4 weeks; range 27.4–35.1 weeks). All analyses were adjusted for gestational age and estimated fetal weight at the time of measurement

*PI* pulsatility index, *PIV* pulsatility index for veins, *PSV* peak systolic velocity, *NS* not significant

^a^Cardiac measurements were additionally adjusted for fetal heart rate

### Afterload

The pulsatility index of the umbilical artery was significantly lower in male fetuses compared to female fetuses (difference −0.03, 95 % CI −0.05; −0.01, *p* < 0.05). No significant differences regarding the pulsatility index of the middle cerebral artery were observed between male fetuses and female fetuses. In male fetuses, a significant lower peak systolic velocity was observed in the pulmonary artery (difference −1.33, 95 % CI −2.63; −0.03, *p* < 0.05), with a similar, but not significant, trend towards a lower peak systolic velocity of the aorta ascendens.

## Discussion

Though sex divergences in cardiovascular health in adulthood have been intensively studied, to date, sex-based differences focusing on the early phases of life have received little attention. We describe differences between male and female fetuses in several cardiovascular compartments. In male fetuses, the contribution of the early ventricular filling phase is mainly passive, and hence, the E/A ratio is lower with a decreased umbilical PI and increased PIV of the ductus venosus. While it is difficult to relate these measurements to exact cardiac function, our findings strongly suggest that the known differences in cardiovascular performance between the sexes already start in utero.

### Preload

Ventricular filling or diastole is an essential but complex mechanism. Assessing diastolic function is difficult as it is influenced by many factors such as heart rate, loading conditions, myocardial mass, and myocardial relaxation [11]. Additionally, diastolic features may evolve continuously throughout life. In fetuses, the majority of diastolic filling occurs during atrial contraction [12]. It is only postnatally that the contribution of passive filling increases towards a characteristic E wave predominance as seen in healthy young adults [12–15]. In male fetuses, we observed a reduced E/A ratio for both atrioventricular valves which was mainly caused by lower E wave velocities. It seems that ventricular relaxation contributes less to the ventricular filling phase in male fetuses as compared to female fetuses despite a lower heart rate and at a consequently longer filling time. The increased pulsatility index in the ductus venosus further suggests that this is accompanied by an increased filling pressure or preload [16]. Similar changes are observed in growth-restricted fetuses [17]. It remains difficult however, based on our observations, to evaluate whether this pattern reflects a healthy physiological fetal pattern responding to a different preload or that it represents a slightly reduced ventricular relaxation due to increased ventricular stiffness. In both children and adults, diastolic function is best assessed by combining mitral inflow patterns with tissue Doppler. This provides more load-independent information, and it better reflects myocardial relaxation thereby allowing differentiation between a normal and a pseudonormal pattern. However, in fetuses, tissue Doppler is still experimental and measuring loading conditions remains difficult. Therefore, it is challenging to relate a fetal Doppler pattern to exact fetal diastolic function [18–20]. Nevertheless, our data clearly demonstrate significant differences in the maturation of ventricular filling between male and female fetuses. Further evaluation of these results with follow-up after delivery is required to confirm and extend these findings.

### Afterload

The pulmonary artery is the systemic artery of the fetal circulation. Only a minority of its output flows to the lungs; the rest flows into the descending aorta. We observed a decreased peak systolic velocity of the pulmonary artery along with a lower pulsatility index of the umbilical artery in male fetuses. The left ventricle mainly supplies the fetal head and upper extremities. Similarly to the pulmonary and umbilical artery, we observed a non-significant trend towards a lower peak systolic velocity in the aorta and a reduced pulsatility index of the middle cerebral artery. Despite slight differences in estimated fetal weight, there were no differences in cardiac output of either ventricles, indicating similar systolic function between both sexes. Similar findings were reported by Prior et al. [21] who reported on a lower middle cerebral artery pulsatility index, middle cerebral artery peak velocity, and umbilical venous flow/kilogram in male fetuses. The observed decreased pulsatility index of the umbilical and middle cerebral artery together with the lower peak velocities in the aorta and pulmonary artery suggest a reduced afterload in male fetuses compared to female fetuses. This allows for a similar output at lower peak velocities. The reduced afterload and the increased fetal weights suggest a reduced placental resistance and/or enhanced arterial compliance in male fetuses. However, it might just be that male fetuses exhibit a larger aortic and pulmonary valve diameter with a lower peak systolic velocity as a result. To test this, we added aortic and pulmonary artery size to our statistical models. However, this did not affect the results.

### Pathway

Our results suggest the existence of intracardiac hemodynamic differences between male fetuses and female fetuses measurable from 30 weeks gestation onwards. This phenomenon is also observed in later life and could explain the differences in cardiovascular risk profiles between men and women at various age categories. Premenopausal women exhibit a lower incidence of CVD and hypertension compared with age-matched men [22]. The overall change in risk after menopause suggests a regulatory role for estrogens in the maintenance of vascular function and structure. This is confirmed by studies showing a normalizing effect of ovariectomy-induced high blood pressure after 17β-estradiol replacement and studies that show differences in female vascular function in relation to menstrual cycle and estrogen concentrations [23]. Furthermore, studies using rat castration models show that castration reduces hypertension and that this effect is reversed by testosterone replacement. This suggests an important role for both male and female sex hormones on vascular function [24]. The vascular endothelium is important in the control of vascular tone and in the regulation of peripheral blood pressure [25]. It maintains vascular homeostasis through the release of active vasodilators encompassing nitric oxide (NO), prostacyclin, and endothelium-derived hyperpolarizing factor (EDHF). Previously, it was shown that testosterone inhibits the function and production of these vasodilators with a decrease in endothelium-dependent vasorelaxation [26–28]. Reyes et al. [29] were able to measure differences in testosterone in the serum of 46 male fetuses and 33 female fetuses delivered by hysterotomy from already 10 weeks gestation onwards. From this, we hypothesize that our observed differences in cardiovascular function between male fetuses and female fetuses could, at least partially, be caused by a testosterone-mediated-endothelium response. In this respect, Chinnathambi et al. [30] showed that prenatal testosterone exposure leads to an increase in blood pressure associated with blunting of endothelial cell-associated relaxation. However, it might also be that the hypothesized differences in endothelial function are secondary to arterial pressure alterations.

Arterial compliance in fetal life may be influenced by a decrease in elastin deposition, a major determinant of stiffness, caused by altered hemodynamic conditions or changed blood pressure [31]. Blood flow is an important hemodynamic stimulus for arterial wall development.

Hemodynamic disturbances in fetal growth are known to result in permanent fetal cardiovascular changes like increased arterial wall stiffness and left ventricular dysfunction [31–34]. The changes seem to persist later in life. Similar to this, our results show some consistencies with these phenomena, indicating that fetal circulation differences between male fetuses and female fetuses might underlie the differences in cardiovascular risk profiles between men and women in later life.

### Strengths and limitations

The main strength of our study is the prospective design from early fetal life onwards within a large population-based cohort. We had a large sample size of 1028 subjects. Of all mothers of the Generation R Study who were approached for the detailed subgroup, 80 % participated in the focus study. Non-participation was mainly due to lack of time. No differences in offspring birth weight and maternal characteristics were found between mothers participating and not participating in the Generation R Focus Study [7, 8]. Furthermore, the Generation R Focus Study is ethnical homogeneous. Thus, we do not assume major health-related differences between these participants and non-participants. To our knowledge, this is the largest population-based cohort study in which such detailed fetal circulation variables were established. The population-based setting enabled us to assess fetal circulation physiology unbiased over the whole range of estimated fetal weight, rather than in fetuses with growth restriction or other pregnancy-related complications only [6].

## Conclusions

Cardiovascular function in male fetuses is different than cardiovascular function in female fetuses with increased preload but reduced afterload in male fetuses. These fetal circulation differences might contribute to the sex-specific cardiovascular risk profiles in adult life. Follow-up studies in our children are currently performed to examine whether and to what extent differences in fetal circulation hemodynamics persist during childhood and whether they are related to cardiac function and blood pressure development in postnatal life.

## Abbreviations

BMI: Body mass index
CI: Confidence interval
CVD: Cardiovascular disease
EDHF: Endothelium-derived hyperpolarizing factor
NO: Nitric oxide
NS: Not significant
PI: Pulsatility index
PIV: Venous pulsatility index
PSV: Peak systolic velocity
SD: Standard deviation
TVI: Time velocity integral
